# The Lysine Methylase SMYD3 Modulates Mesendodermal Commitment during Development

**DOI:** 10.3390/cells10051233

**Published:** 2021-05-18

**Authors:** Raffaella Fittipaldi, Pamela Floris, Valentina Proserpio, Franco Cotelli, Monica Beltrame, Giuseppina Caretti

**Affiliations:** 1 Department of Biosciences, University of Milan, Via Celoria 26, 20133 Milan, Italy; raffaella.fittipaldi@gmail.com (R.F.); pamela.floris@unimib.it (P.F.); valentina.proserpio@gmail.com (V.P.); franco.cotelli@unimi.it (F.C.); monica.beltrame@unimi.it (M.B.); 2 Candiolo Cancer Institute, FPO-IRCCS, 10060 Candiolo, Italy

**Keywords:** embryonic stem cells, SMYD3, zebrafish, development

## Abstract

SMYD3 (SET and MYND domain containing protein 3) is a methylase over-expressed in cancer cells and involved in oncogenesis. While several studies uncovered key functions for SMYD3 in cancer models, the SMYD3 role in physiological conditions has not been fully elucidated yet. Here, we dissect the role of SMYD3 at early stages of development, employing mouse embryonic stem cells (ESCs) and zebrafish as model systems. We report that SMYD3 depletion promotes the induction of the mesodermal pattern during in vitro differentiation of ESCs and is linked to an upregulation of cardiovascular lineage markers at later stages. In vivo, *smyd3* knockdown in zebrafish favors the upregulation of mesendodermal markers during zebrafish gastrulation. Overall, our study reveals that SMYD3 modulates levels of mesendodermal markers, both in development and in embryonic stem cell differentiation.

## 1. Introduction

Epigenetic regulation of gene expression plays a pivotal role in the establishment of developmental programs and the maintenance of the differentiated state [1]. During development, transcription factors and chromatin modifiers are either activated or repressed to commit specific cells to defined lineages in response to external cues [2].

Pluripotent embryonic stem cells (ESCs) possess the unique ability to self-renew and to commit towards differentiation of several lineages [3]. Due to this feature, they represent a powerful tool that became an in vitro system to study molecular mechanisms underlying lineage commitment and to model embryonic development. The maintenance of pluripotency is endowed with the interconnected circuitry of the pluripotency transcription factors OCT4, NANOG and SOX2. These core transcription factors concurrently recruit several chromatin regulators to the genomic regions of stemness genes to maintain their transcriptional activation and sustain the repression of differentiation genes through repressive epigenetic complexes [4,5]. Several chromatin modulators such as Polycomb and MLL complexes, MOF and JMJ proteins, have been described as pivotal regulators of stem cell biology [6,7,8]. Some of these factors are under the direct transcriptional control of the core pluripotency network, controlled by OCT4, NANOG, and SOX2 [9]. Two reports have revealed SMYD3 as an OCT4/NANOG/SOX2-regulated gene in mouse embryonic stem cells [10,11], hinting for a possible role for SMYD3 in ESC biology. SMYD3 belongs to the SMYD family of methylases, which is composed of five members. SMYD proteins share the unique architecture of their SET domain, which is split into two parts by a MYND (myeloid translocation protein 8, nervy, DEAF-1) domain and followed by a cysteine-rich post-SET domain. SMYD proteins are expressed in the cardiac muscle during development and in striated muscle [12,13,14,15,16]. Other SMYD family members have been shown to play a role during development [12]. *Smyd1* deletion in mice disrupts normal maturation of ventricular cardiomyocytes and right ventricular development [14,17]; *smyd1* knockdown in zebrafish (*Danio rerio*) embryos results in absence of heartbeat and disrupted myofibril organization and *smyd1a/smyd1b* double mutants show complete disruption of sarcomere organization in fast and slow muscles [18,19,20]. *smyd2a* zebrafish morphants show an impaired myofibril organization in cardiac muscles [21]. Moreover, *smyd2a* morphants display a delay in development and tail abnormalities. This phenotype correlates with reduced expression of *ntl* and induction of Nodal-related genes at gastrulation. In vitro experiments in human ESCs show that SMYD2 is induced during differentiation and its depletion affects endodermal markers [22,23]. Drosophila *dSmyd4* knockdown suggests a role for dSmyd4 in development or function of adult muscles [16]. SMYD5 negatively regulates lineage-specific genes and plays a role in the maintenance of self-renewal in mESCs [24] and in hematopoiesis during zebrafish embryogenesis [25].

SMYD3 is expressed in the mouse embryo [26] and it becomes over-expressed in several tumors where it promotes invasiveness, proliferation and metastasis [26,27,28,29,30] and multiple reports have focused on the role of SMYD3 role in promoting tumorigenesis [31,32].

Despite its expression in normal tissues, very little is known about SMYD3 physiological function. Insights into the role of SMYD3 in development were provided by a report by Fujii et al., showing that Smyd3 depletion correlates with heart and skeletal muscle abnormalities in zebrafish embryos [33]. Moreover, SMYD3 regulates mouse skeletal muscle cells differentiation [34] and myotube size in atrophic conditions [35]. Defects in cardiac morphogenesis of *smyd3* morphants were also confirmed by Kim et al. [36].

Here, we employed ESCs as a model to recapitulate embryonic development and found that SMYD3 depletion results in upregulation of mesendodermal markers during the formation of embryoid bodies (EBs). To parallel these findings with an in vivo approach, we employed zebrafish, in which early development is well characterized, rapid, external, and at the same time it is orchestrated by molecular determinants conserved in mammals. Due to Smyd3 involvement in cardiomyocyte differentiation and myogenesis in zebrafish [32], we differentiated ESCs towards the cardiovascular lineage and investigated the behavior of markers of early cardiac differentiation, following SMYD3 depletion.

Collectively, our results show that SMYD3 plays a role in mesendoderm commitment, during the differentiation towards distinct germ layers fates.

## 2. Materials and Methods

### 2.1. Cell Cultures

All cell lines were obtained from ATCC and were cultured at 37 °C with 5% CO_2_ in an incubator with humidified atmosphere. Undifferentiated R1 mouse embryonic stem cells (CVCL_2167) were plated on mitomycin C-treated mouse embryonic fibroblast (CF-1-MEF, CVCL_5251 ) feeders for 2 passages and then plated on tissue culture dishes coated with 0.1% gelatin (Millipore, Milan, Italy), in DMEM (LONZA, Milan, Italy) supplemented with 15% fetal bovine serum (HyClone, Milan, Italy), 1% non-essential amino acids (NEAA) (GIBCO, Monza, Italy), 1% penicillin/streptomycin (P/S) (GIBCO, Monza, Italy), 1% Glutamax (GIBCO, Monza, Italy), 0.1 mM β-mercaptoethanol (Sigma, Milan, Italy) and 1000 U/mL leukemia inhibitory factor LIF (Millipore). ESC cells were used for maximum 10 passages.

HEK-293T (CVCL_0063) cells used for virus production, NIH3T3 (CVLC_0594), C2C12(CVCL_0188), and MEF cells used for ES feeder were cultured in DMEM (LONZA, Milan, Italy), containing 10% FBS (GIBCO, Monza, Italy), 1% P/S (Invitrogen, Monza, Italy); medium was supplemented with 100 uM β-mercaptoethanol (Sigma, Milan, Italy) for MEF cells.

### 2.2. In Vitro Differentiation of ES Cells

To induce EB formation, undifferentiated ES cells were cultured in hanging drops for 3 days (d) at a density of 800 cells/20 μL of differentiation medium (DM), which consisted of DMEM supplemented with penicillin/streptomycin, 0.1 mM nonessential amino acids, 0.1 mM 2-βmercaptoethanol and 10% FBS (LONZA, Milan, Italy). EBs were transferred to suspension cultures for an additional 3 d (d3 + 3) in non-adherent Petri dishes. For cardiac differentiation of ESCs, EBs were plated in differentiation medium in 0.1% gelatin (Millipore, Milan, Italy) coated plates. The medium was changed every two days.

### 2.3. Zebrafish Lines and Maintenance

Zebrafish (*Danio rerio*) embryos were raised and maintained under standard conditions and national guidelines (Italian decree 4 March 2014, n.26). No Ethics Committee approval is needed for the use of Zebrafish embryos at the stage employed, according to the Italian law. Zebrafish AB wild-type fish were obtained from the Wilson lab, University College London, London, UK. For in vivo observations and imaging, embryos beyond 24 hpf were washed, dechorionated and anaesthetized with 0.016% tricaine (ethyl 3-aminobenzoate methanesulfonate salt; Sigma-Aldrich, Milan, Italy) before observations and picture acquisition. Embryos were staged according to morphological criteria [37].

### 2.4. Whole Mount In Situ Hybridization, Embryo Sectioning and Imaging

Whole mount in situ hybridizations were carried out as described [38]. The riboprobes were synthesized using the Ambion^®^ MAXIscript^®^ SP6/T7 in vitro Transcription Kit (Thermo Fisher Scientific, Monza, Italy). Images of stained embryos were taken on a Leica MZFLIII epifluorescence stereomicroscope equipped with a DFC 480 digital camera and LAS Leica imaging software v4.1 (Leica, Wetzlar, Germany).

### 2.5. Morpholino Microinjection

Antisense morpholinos were purchased from Gene Tools (LLC, Philomath, OR, USA). Morpholinos were diluted in Danieau solution [39] and injected into 1-to-2 cell-stage embryos using Eppendorf FemtoJet Micromanipulator 5171. Rhodamine dextran (Molecular Probes, Life technology, Monza, Italy) was co-injected as dye tracer. *smyd3* knockdown was performed injecting 0.2–0.4 pmol/embryo of a translation blocking MO (*smyd3*-MO 5′-CCTCTCCATAATCACAGCCTCCATC-3′), previously described [33]. A standard control oligo (std-MO, 5′-CCTCTTACCTCAGTTACAATTTATA-3′), with no target in zebrafish embryos, was also used to check for non-specific effects due to the injection procedure.

### 2.6. Virus Production and Plasmids

mESC cells were infected with viral supernatant produced as follows: HEK293T was transiently transfected with pCL-ECO (pCL-Eco was a gift from Inder Verma (Addgene plasmid #12371; http://n2t.net/addgene:12371, accessed on 17 February 2021; RRID: Addgene_12371) and retroviral vector pSh-Scramble or pSh-SMYD3 by using the calcium phosphate technique. ShRNA for SMYD3 or a scrambled sequence was cloned in a pSuper-retro-puro retroviral vector that was previously described [35]. After 10 h, the medium was changed, harvested 36 h after transfection, and used to infect mES cells in the presence of 4 μg/mL polybrene (Sigma). We performed spinoculation at 4000 rpm for 45 min. Infected cells were selected with 1 μg/mL puromycin (Sigma) for 3 days.

HEK293T was transfected with psPAX2 and pMD2.G (gifts from Didier Trono, Addgene plasmid #12259, 12260; http://n2t.net/addgene:12259, accessed on 17 February 2021; RRID: Addgene_12259; http://n2t.net/addgene:12260, accessed on 17 February 2021; RRID: Addgene_12260) and pLKO-puro-sh_Scramble or lentivirus T/Brachyury-eGFP. T/Brachyury-eGFP Rex Neo was a gift from Mark Mercola (Addgene plasmid #21222; http://n2t.net/addgene:21222, accessed on 17 February 2021; RRID: Addgene_21222) [40].

### 2.7. Reverse Transcriptase PCR and Real-Time PCR

Total RNA was isolated by the TriReagent (SIGMA) method and reverse transcribed to cDNA with High Capacity cDNA Reverse Transcription Kit (Life Technologie, Monza, Italy) and subjected to qPCR analysis [41]. Sequences of oligonucleotides used in qRT-PCR are reported in Table 1 and were obtained from previous reports (see References in the table) or designed with the Primer3 software v0.4.0 and checked with the UCSC in silico PCR tool. Quantitative PCR was performed using SYBR green IQ reagent (GeneSpin, Milan, Italy) in the iCycler IQ detection system (Biorad, Milan, Italy). qPCR was performed in triplicate for each of the biological replicates. Data were analyzed using the delta-delta Ct method.

### 2.8. Protein Extracts

Total cell extracts were prepared lysing the cells pellet in RIPA buffer as described [35]. The extracts were centrifuged at maximum speed for 15 min and the supernatant was collected.

Protein concentration was determined by Bradford assay. Total cell extracts were separated by SDS-PAGE and immunoblotted with specific antibodies (SMYD3: GeneTex, GTX121945, GAPDH: Santa Cruz, sc-32233, NANOG Cosmobio REC-RCAB002P-F, OCT4: ab19857, SMA: ab15694, FLK: Novus NB100-627SS).

To isolate chromatin, samples were prepared following Mendez’s protocol described in [51]. Briefly, cells were resuspended in buffer A (10 mM HEPES pH 7.9, 10 mM KCl, 1.5 mM MgCl_2_, 0.34 M sucrose, 10% glycerol, 1 mM DTT and Protease inhibitor cocktail). Triton X-100 (0.1%) was added, and the cells were incubated for 5 min on ice. Nuclei were collected in pellet 1 (P1) by low-speed centrifugation. The supernatant (S1) was further clarified by high-speed centrifugation to remove cell debris and insoluble aggregates. Nuclei were washed once in buffer A, and then lysed in buffer B (3 mM EDTA, 0.2 mM EGTA, 1 mM DTT, protease inhibitors). Insoluble chromatin was collected by centrifugation, washed once in buffer B, and centrifuged again under the same conditions. The final chromatin pellet (P3) was resuspended in Laemmli buffer and sonicated.

### 2.9. Immunofluorescence Analysis

Immunostaining of cultured cells was carried out as previously described [49]. Briefly, the cells were fixed for 10 min in 4% paraformaldehyde (PFA) in PBS and permeabilized with 0.2% Triton X-100 in PBS for 10 min. After incubation in 3% BSA for at least 20 min at room temperature, cells were incubated for 2 h at room temperature with antibodies. After washing the samples with 0.1% Triton X-100 in PBS for 10 min, cells were incubated with secondary antibodies, which are fluorescently labeled. DAPI was used for nuclear staining. The samples were examined with a fluorescence microscope (Carl Zeiss, Varese, Italy). Pictures of staining were obtained using an AxioCam (Carl Zeiss, Varese, Italy).

### 2.10. Statistical Analysis

Data are represented as mean ± standard error of the mean (SEM). Data are analyzed by using a two-way ANOVA followed by post hoc Bonferroni’s test comparison. Probability values of less than 0.05 were considered statistically significant.

## 3. Results

### 3.1. SMYD3 Is Expressed in the Developing Mouse Embryo and in Embryonic Stem Cells

**Figure 1:**
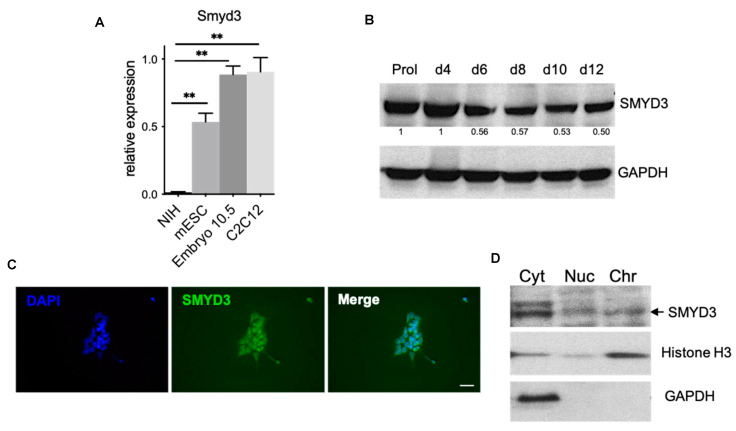
SMYD3 is expressed in mouse embryo and in mouse embryonic stem cells (mESCs). (**A**) mRNA was extracted from NIH3T3, C2C12 myoblasts and mESC in parallel with d10.5 embryo and *Smyd3* transcript levels were measured by qRT-PCR. Data were normalized to GAPDH. Means from three independent experiments are shown. ** *p* < 0.01 value for the significance is shown in the plot. (**B**) Immunoblot analysis of SMYD3 protein levels at different time points of ESC differentiation. GAPDH served as a loading control. Normalized band intensity in immunoblots is reported below signals. Representative image of three independent experiments. (**C**) Immunofluorescence was performed on undifferentiated mESCs with an antibody raised against SMYD3. Nuclei were visualized by DAPI staining (blue). Scale bar: 50 μm. (**D**) ESCs were analyzed by biochemical fractionation, followed by immunoblot, to characterize SMYD3 distribution in different fractions: cytoplasmic or soluble fraction (Cyt), solubilized nuclear proteins fraction (Nuc), and chromatin-nuclear matrix-bound fraction (Chr). GAPDH served as Cyt control, while H3 served as Chr control. Representative image of two independent experiments.

SMYD3 functions have been mainly studied in different cancer cell lines, where it promotes proliferation and invasion [26,52]. To investigate if SMYD3 may play a role in development, we first analyzed Smyd3 expression levels in non-tumor derived cells. *Smyd3* transcripts were high in mESCs and C2C12 myoblasts [34], when compared to the fibroblast cells NIH3T3. *Smyd3* mRNA was also expressed in the d10.5 mouse embryo (Figure 1A).

To investigate SMYD3 role during development, we used mESCs as a model system and we first analyzed SMYD3 protein levels by Western blot throughout differentiation of EBs and observed that SMYD3 levels decrease at early stages of differentiation, and they maintain constant till day 12 (Figure 1B).

We also checked SMYD3 distribution in different intracellular compartments by chromatin fractionation and immunofluorescence assays. These experiments revealed that SMYD3 is expressed in mESCs and it is detectable in the cytoplasm, nuclei and chromatin fraction, as previously shown for cancer cell lines [28] (Figure 1C,D).

Together, these data indicate that SMYD3 is expressed in mESC and EBs, which can be used to further investigate SMYD3 role in stemness and lineage commitment.

### 3.2. SMYD3 Depletion Does Not Affect Stemness

**Figure 2:**
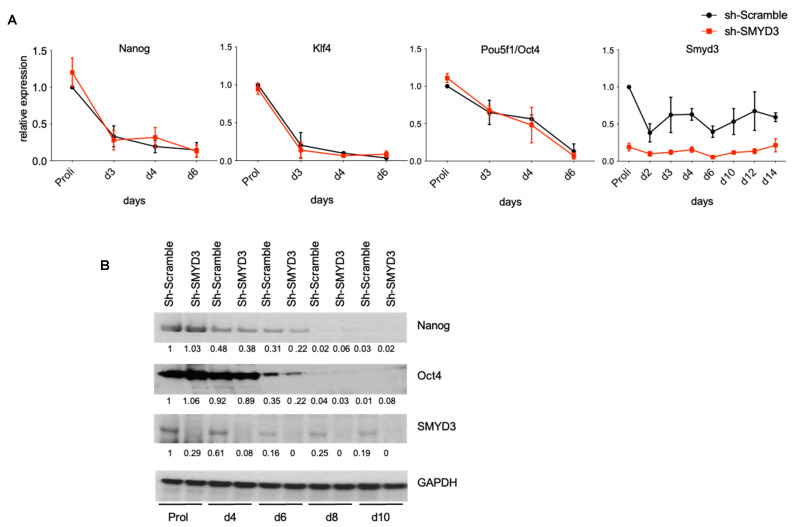
SMYD3 depletion does not interfere with stemness. (**A**) mRNA was extracted at different stages of ESC differentiation, and *Smyd3* or pluripotency-related gene (*Nanog, Klf4, Pou5f1/Oct4*) transcript levels were evaluated throughout differentiation by qRT-PCR. Means and SEM from three independent experiments are shown. (**B**) Nanog, Oct4 and SMYD3 protein levels were analyzed by immunoblot. Data were normalized to GAPDH. Normalized band intensity in immunoblots is reported below signals. Representative image of three independent experiments.

To test if SMYD3 plays a role in mESCs differentiation, we performed loss-of-function experiments by reducing SMYD3 levels with a short hairpin RNA (shRNA) targeting Smyd3 and establishing the stable cell line Sh-SMYD3 depleted of SMYD3, which was compared to control Sh-Scramble cells (Figure 2A). Pluripotency markers *Nanog*, *Klf4* and *Pou5f1/Oct4* did not change their expression level in SMYD3-depleted and control Sh-Scramble cells at different stages of differentiation.

These results were confirmed by immunoblot analysis, with antibodies raised against NANOG and OCT4 in proliferating mESCs and after 4–6–8–10 days of differentiation through EB formation and maintenance (Figure 2B). Overall, we concluded that SMYD3 does not play a role in mESC stemness maintenance.

### 3.3. SMYD3 Depletion Accelerates Mesendoderm Marker Expression in mESCs

**Figure 3:**
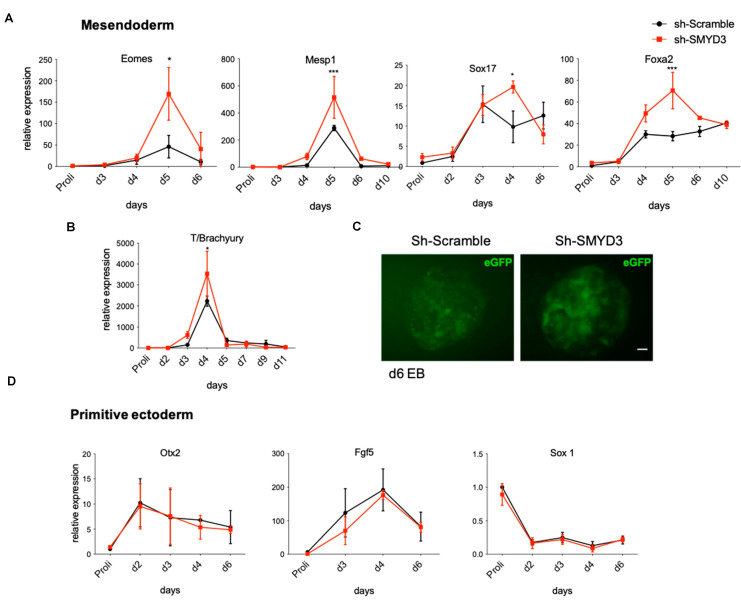
SMYD3 knockdown increases mesendodermal marker expression in differentiating mESCs. (**A**,**B**) mRNA levels of mesendodermal markers (*Eomes*, *Mesp1*, *Sox17*, *Foxa2*, *T/Brachyury*) were measured by qRT-PCR. Data were normalized to *Gapdh*. Means and SEM from three independent experiments are shown. Significance was calculated by 2-way Anova, followed by Bonferroni post hoc test. * *p* < 0.05, *** *p* < 0.001 value for the significance is shown in the plot. (**C**) eGFP reporter fluorescence was analyzed in EBs infected with Sh-Scramble/Brachyury-eGFP and Sh-SMYD3/Brachyury-eGFP at day 6 of differentiation. Scale bar: 20 μm. (**D**) Primitive ectoderm genes (*Otx2*, *Fgf5*, *Sox1*) expression was measured by qRT-PCR at different time points. Means and SEM from three independent experiments are shown.

Next, we sought to elucidate whether SMYD3 regulates the differentiation potential of mESCs. We examined and compared mRNA expression patterns for various lineage markers at different time points following LIF (leukemia inhibitory factor) withdrawal and EBs formation. As expected, the mouse mesoendoderm markers, *Eomes*, *Mesp1*, *Sox17*, *Foxa2,* were upregulated in Sh-Scramble control cells and peaked through day 4–5 of differentiation. Strikingly, in Sh-SMYD3 cells, the expression of *Eomes*, *Mesp1*, *Sox17* and *Foxa2* was significantly higher when compared to the control (*p* < 0.05) (Figure 3A).

To further characterize the role of SMYD3 in the early stages of lineage commitment, we analyzed the pan-mesodermal marker *T/Brachyury* expression level, which was upregulated in SMYD3-depleted cells (Figure 3B). Moreover, we co-infected undifferentiated Sh-SMYD3 or Sh-Scramble mESCs with a virus carrying eGFP cDNA under the control of the *T/Brachyury* promoter. In Sh-SMYD3 cells derived EBs, eGFP fluorescence peaked at day 6 of differentiation, and GFP positive cells foci were more abundant than in Sh-Scramble-derived EBs (Figure 3C).

Conversely, markers of the primitive ectoderm *Fgf5* and *Otx2* as well as the *Sox1* ectoderm marker behaved in a similar manner in SMYD3 knocked-down Sh-SMYD3 and Sh-Scramble control cells (Figure 3D). Overall, these data show that SMYD3 depletion favors mesendodermal commitment in differentiating EBs and does not affect primitive ectoderm specification.

### 3.4. smyd3 Knockdown Promotes Mesendodermal Fate during Zebrafish Gastrulation

To test whether our findings were confirmed in an in vivo model, we injected *smyd3* morpholino (*smyd3*-MO) in zebrafish embryos, at the 1–2 cell stage. Morphants displayed curved trunks, pericardial edema and impaired circulation compared to the control embryos. Embryos were divided in four phenotypic classes, with increasing severity (Supplementary Figure S1A,B), and their defects and severity reflected the previously reported phenotype for *smyd3* morphants [33,53].

**Figure 4:**
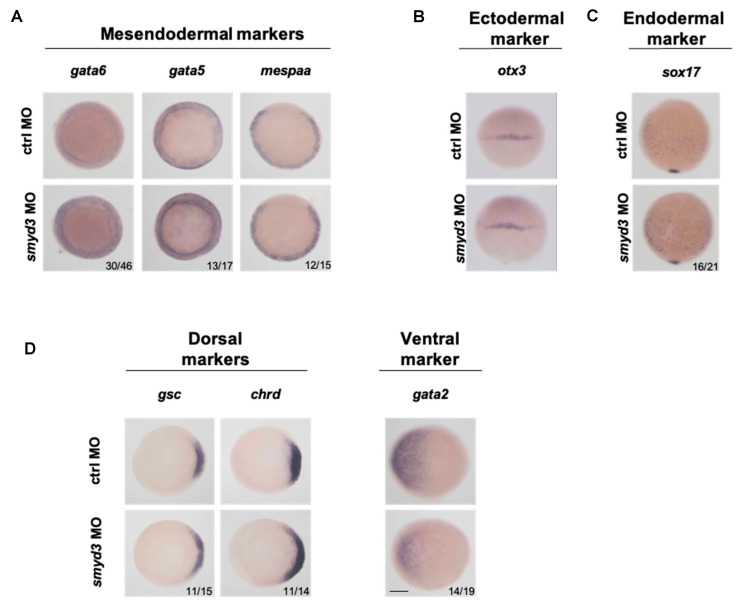
*smyd3* knockdown promotes mesendodermal fate in zebrafish. (**A**) Expression of the mesendodermal markers, *gata6*, *gata5* and *mespaa*, was evaluated by ISH in *smyd3* morphants at 50% of epiboly, in comparison to standard control embryos. Embryos are shown in dorsal view. (**B**) ISH of the ectodermal marker *otx3* was performed in morphants and standard control embryos at 50% epiboly stage. (**C**) The endodermal marker *sox17* was examined at 75% epiboly and shown in dorsal view. (**D**) ISH of dorsal mesodermal markers *gsc*, *chrd* and of ventral mesodermal marker *gata2* was performed at 50% epiboly and shown in dorsal view. Scale bar: 250 μm.

To analyze zebrafish mesendodermal markers in *smyd3* morphants, we performed in situ hybridization (ISH) at the onset of gastrulation (50% epiboly) and analyzed the expression of the *Mesp1* ortholog *mespaa* and of additional markers that play a role in mesendodermal specification. We found that the *gata6*, *gata5*, and *mespaa* signals were enhanced in the blastodermal margin in *smyd3* morphants (Figure 4A).

Conversely, the expression of the ectodermal marker *otx3* was comparable in *smyd3* morphant and standard control embryos (Figure 4B). In mid-gastrulation (75% epiboly), *sox17* levels were enhanced in endodermal cells of *smyd3* morphants (Figure 4C), in agreement with that observed in ESCs (Figure 3A). Additionally, the dorsal mesodermal markers goosecoid (*gsc*) and chordin (*chrd*) showed an expansion in the blastodermal margin of *smyd3* knocked down zebrafish embryos (Figure 4D), while the signal for the ventral mesodermal marker *gata2* was reduced. Overall, these data suggest that *smyd3* knockdown favors the induction of mild mesendodermal fate in zebrafish embryos, supporting the in vitro results observed in mESCs.

### 3.5. SMYD3 Knockdown Influences Later Stages of Differentiation

SMYD3-depleted mESCs appeared to be more prone to differentiate towards the mesendodermal lineage (Figure 3). To test whether upregulation of mesendodermal markers persisted at later stages of differentiation, we differentiated Sh-SMYD3 and Sh-Scramble EBs toward cardiovascular fate. Differentiation toward this lineage was selected because of the possible role of SMYD3 in cardiomyocyte differentiation and the cardiac phenotype observed in zebrafish [33].

**Figure 5:**
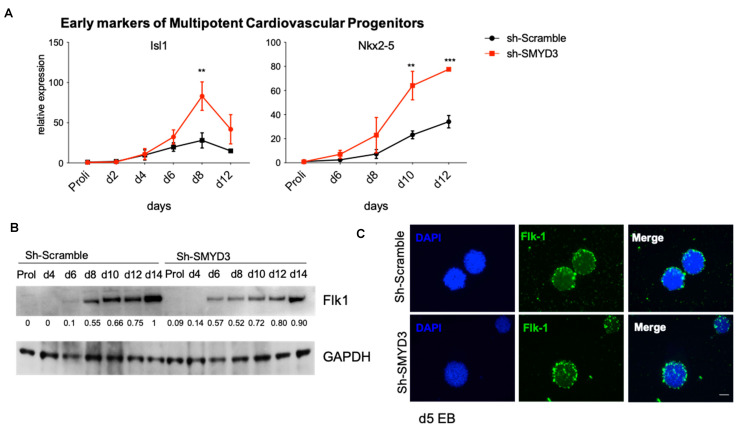
SMYD3 depletion enhances early cardiac differentiation in mESCs. (**A**) Gene expression of early cardiac markers *Isl1* and *Nkx2-5* was analyzed by qRT-PCR. Data were normalized to *Gapdh*. Means and SEM from three independent experiments are shown. Significance was calculated by 2-way Anova, followed by Bonferroni post hoc test. ** *p* < 0.01, *** *p* < 0.001 value for the significance is shown in the plot. (**B**) Western blot analysis was performed on mESCs protein extracts at different time of differentiation, with antibodies raised against Flk1. GAPDH serves as a loading control. Normalized band intensity in immunoblots is reported below signals. (**C**) Immunofluorescence analysis was performed with Flk1 antibodies at d5 of EB differentiation. DAPI stains nuclei. Scale bar: 200 μm.

Early markers of multipotent cardiovascular progenitors such as *Nkx2-5* and *Isl1* were more robustly expressed in SMYD3-depleted EBs when compared to Sh-Scramble control EBs (Figure 5A).

In parallel, we investigated the expression of a second mesodermal marker, Kdr/Flk1. Kdr/Flk1 protein expression in SMYD3 depleted Sh-SMYD3 EBs was anticipated from day 8 to day 6, when compared to Sh-Scramble control cells (Figure 5B). This finding was confirmed by immunofluorescence experiments in Sh-SMYD3 and control EBs at day 5 of differentiation. At this stage, Flk1-positive cells were more abundant in SMYD3-depleted EBs, when compared to control Sh-Scramble cells, in immunofluorescence assays (Figure 5C).

**Figure 6:**
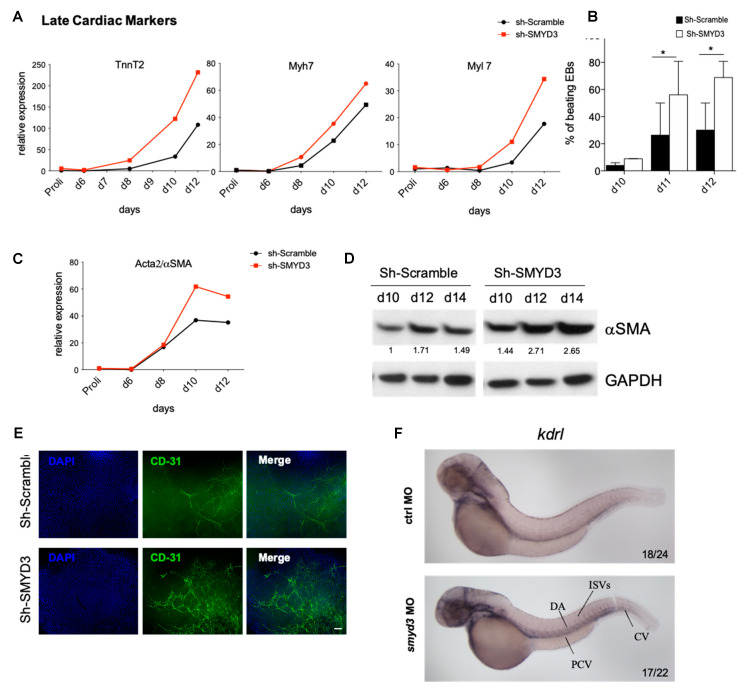
SMYD3 modulates late cardiovascular markers. (**A**) Cardiac markers, TnnT2, Myh7 and Myl7, were measured by qRT-PCR at d0, d6, d8, d10 and d12 of mESCs differentiation. Data were normalized to GAPDH. One representative experiment is shown; *n* = 2. (**B**) Beating EBs were counted at d10, d11, d12 of EBs formation in Sh-Scramble and Sh-SMYD3. Significance was calculated by 2-way Anova, followed by Bonferroni post hoc test. * *p* < 0.05 value for the significance is shown in the plot. *n* = 3. (**C**) αSMA mRNA levels were measured by qRT-PCR as in (**A**). One representative experiment is shown: *n* = 2. (**D**) Western blot analysis of αSMA protein extracts of Sh-Scramble and Sh-SMYD3 EBs at d10, d12, d14. GAPDH serves as a loading control. Normalized band intensity in immunoblots is reported below signals. (**E**) Immunofluorescence microscopy was performed on d10 EBs with antibodies against the endothelial marker CD31. DAPI stains nuclei. Scale bar: 100 μm. (**F**) *kdrl* expression was analyzed at 48 hpf, in standard control and *smyd3* morphants. Lines show posterior cardinal vein, dorsal aorta, inter-segmental vessels and caudal vein (PCV, DA, ISVs, CV, respectively).

In order to investigate whether SMYD3 depletion enhanced the expression of cardiac lineage markers in EBs, we analyzed the transcript levels of late cardiac markers, e.g., *TnnT2*, *Myh7*, and *Myl7*, which are essential components of the contractile cardiac machinery. We detected elevated mRNA levels of *TnnT2*, *Myh7* and *Myl7* in SMYD3 depleted EBs (Figure 6A). This increase was paralleled by a higher percentage of beating EBs in SMYD3 depleted EBs compared to the control ones (Figure 6B).

Since Flk1 positive cells give rise to cardiomyocytes, smooth muscle and endothelial populations, we also explored the expression levels of smooth muscle actin (αSMA) and endothelial marker CD31. *Acta2/αSMA* mRNA and protein levels were increased at d10 of differentiation (Figure 6C,D). The endothelial marker CD31 was also increased in Sh-SMYD3 EBs compared to Sh-Scramble ones, as shown by IF signal density at day 10 (Figure 6E).

Our results show that SMYD3 depletion promotes cardiac, smooth muscle and endothelial marker expression in differentiating EBs.

Additionally, we analyzed the expression of the endothelial marker kinase insert domain receptor like (*kdrl*), which is a *Kdr/Flk1*-related gene in zebrafish. Through in situ hybridization analyses, we observed an increased *kdrl* expression in *smyd3* morphants at 48 hpf (Figure 6F). Enhanced expression was particularly evident in the posterior cardinal vein, caudal vein, dorsal aorta and intersegmental vessels of *smyd3* knocked down embryos, suggesting that the kinase receptor is upregulated in the endothelial cells lacking Smyd3.

Taken together, these findings hint at a role for SMYD3 in modulating the accurate formation of mesodermal lineages.

## 4. Discussion

SMYD3 appears to be regulated by the core pluripotency OCT4/NANOG/SOX2 transcription factor network in proliferating mESCs [10,11], and, in agreement with these findings, we observed that SMYD3 levels tend to decrease at d2 of ESCs differentiation. Following SMYD3 knockdown, we observed an upregulation of several mesendodermal markers at early time points of mESC differentiation. In parallel, an increase in mesendodermal markers was detectable in *smyd3* zebrafish morphants, at gastrulation stages. Since SMYD3 is involved in cardiomyocyte maturation in zebrafish [32,35], we also prompted mESCs to differentiate towards the cardiovascular lineage and found that SMYD3 depletion leads to the upregulation of early and late cardiovascular markers. These data were corroborated by an upregulation of the endothelial marker *kdrl* in *smyd3* zebrafish morphants. Taken together, our results suggest that SMYD3 negatively impacts mesendodermal commitment in mESCs differentiation and in gastrulating zebrafish embryos. In addition, the cardiac phenotype in zebrafish is consistent with the induction of mesendodermal and cardiovascular genes observed in mESC differentiation.

The transcriptional alterations induced by SMYD3 depletion in mESCs appear to be largely dependent on the transcriptional upregulation of *T/Brachyury* and *Eomes* genes, which are key transcription factors for the activation of the mesendodermal fate, acting as repressors of key pluripotency and neuroectodermal genes and as positive regulators of chromatin remodeling at mesendodermal gene enhancers [54].

Employing an experimental design similar to the one presented in this work, Sesè et al. (2013) revealed that SMYD2 regulates endodermal gene transcription, both in human embryonic stem cells (hESCs) and zebrafish [22]. In addition, a more recent report showed that, using a different differentiation protocol, SMYD2 depletion leads to inhibition of *T/BRACHYURY*, *EOMES* and other mesendodermal gene expression in hESC. Furthermore, exogenously expressed SMYD2 is directly recruited to the *T/BRACHYURY* and *EOMES* chromatin regulatory regions [23]. Taken together, these findings suggest that mesendodermal genes may be concurrently regulated by SMYD2 and SMYD3, and the interplay between the two methylases will require further elucidation in future work.

While zebrafish *smyd3* morphants have phenotypic defects, Smyd3 knockout mice do not show an apparent phenotype [55]. We speculate that SMYD3 functions can be partially compensated by other SMYD family members or unrelated proteins during mouse development, and thus SMYD3 loss does not result in any obvious defects in the mouse, and its function may be better characterized by double knock-out experiments with other SMYD family members. Alternatively, SMYD3 functions might be not fully conserved among species.

SMYD3 has both nuclear and cytoplasmic functions, which may depend or not on its methylation activity [53]. We speculate that SMYD3 might participate in the regulation of repressors of the mesendodermal fate. Several signaling pathways modulate mesendoderm formation: FGF and Wnt pathways are required for the proper formation of germ layers [56] and Activin/Nodal and BMP4 pathways further influence mesoderm and endoderm specification [57]. Wnt and Nodal/Activin pathways modulate mesodermal markers transcription [58]. It appears that, in mESCs, SMYD3 may affect one of these pathways to decrease mesendodermal genes transcription.

Recently, SMYD3 was shown to methylate the MAP3K2 and the AKT kinase and affect the Ras-activated pathway in certain types of cancer [55,59]. It is tempting to speculate that SMYD3 modulates other cytoplasmic kinases along the Wnt or Nodal/activin pathway to modulate the outcome of their signaling during development.

Interestingly, SMYD3 localization in ESC is not restricted to the cytoplasm, but it is also present in the nucleus. Therefore, SMYD3 nuclear functions may play a direct role in the chromatin of pivotal regulatory genes, which negatively modulate mesendodermal gene transcription.

## Figures and Tables

**Table 1:** Mouse primers used in quantitative real-time PCR.

GENE		Sequence	Reference
*Nanog*	F R	GCGGACTGTGTTCTCTCAGG CCACCGCTTGCACTTCATCC	[42]
*Klf4*	F R	CGTCCCAGTCACAGTGGTAA AAAAGAACAGCCACCCACAC	[43]
*Eomes*	F R	CCCACGTCTACCTGTGCAAC GGTGGGGTTGAGTCCGTTTA	
*Pou5f1/Oct4*	F R	AAGCAACTCAGAGGGAACCT GGTGATCCTCTTCTGCTTCA	
*Mesp1*	F R	GTTCCTGTACGCAGAAACAGCAT GTTTCTAGAAGAGCCAGCATGTC	[44]
*Sox17*	F R	GCCGATGAACGCCTTTATGGTG TCTCTGCCAAGGTCAACGCCTT	[45]
*T/Brachyury*	F R	TGCCTACCAGAATGAGGAGATTA CCATTACATCTTTGTGGTCGTTT	
*TnnT2*	F R	AGCGCGTGGAGAAGGACCTGA CCGCTCTGCCCGACGCTTT	
*Myh7*	F R	CCAAGGGCCTGAATGAGGAG GCAAAGGCTCCAGGTCTGAG	[46]
*Myl7*	F R	AGGAAGCCATCCTGAGTGCCTT CATGGGTGTCAGCGCAAACAGT	[45]
*Acta2/αSMA*	F R	AGGCACCACTGAACCCTAAG ACAGCACAGCCTGAATAGCC	[47]
*Nkx2-5*	F R	GTCCAGCTCCACTGCCTTCT CAAGTGCTCTCCTGCTTTCC	[48]
*Isl1*	F R	GGCTACACAGCGGAAACACT ACGTGCTTTGTTAGGGATGG	[49]
*Otx2*	F R	CTTCATGAGGGAAGAGGTGGCAC TGGCGGCACTTAGCTCTTCGATTC	
*Fgf5*	F R	GCTGTGTCTCAGGGGATTGT CACTCTCGGCCTGTCTTTTC	[50]
*Smyd3*	F R	AGAGGTGTGCAAGTGATGAAAGT ATCAAATCTTCAATCAGGCTGTG	[35]
*Gapdh*	F R	AACATCAAATGGGGTGAGGCC GTTGTCATGGATGACCTTGGC	[49]

## Supplementary Materials

The following are available online at https://www.mdpi.com/article/10.3390/cells10051233/s1, Figure S1: SMYD3 morphant phenotypic classes.
