# Supplementary material for: The Lysine Methylase SMYD3 Modulates Mesendodermal Commitment during Development
Source: Cells. 2021 May 18;10(5):1233. doi: 10.3390/cells10051233 (PMC8157265; doi:10.3390/cells10051233)
Supplement: Supplementary file 1 [file cells-10-01233-s001.zip › cells-1187132-SI.pptx]

## Slide 1
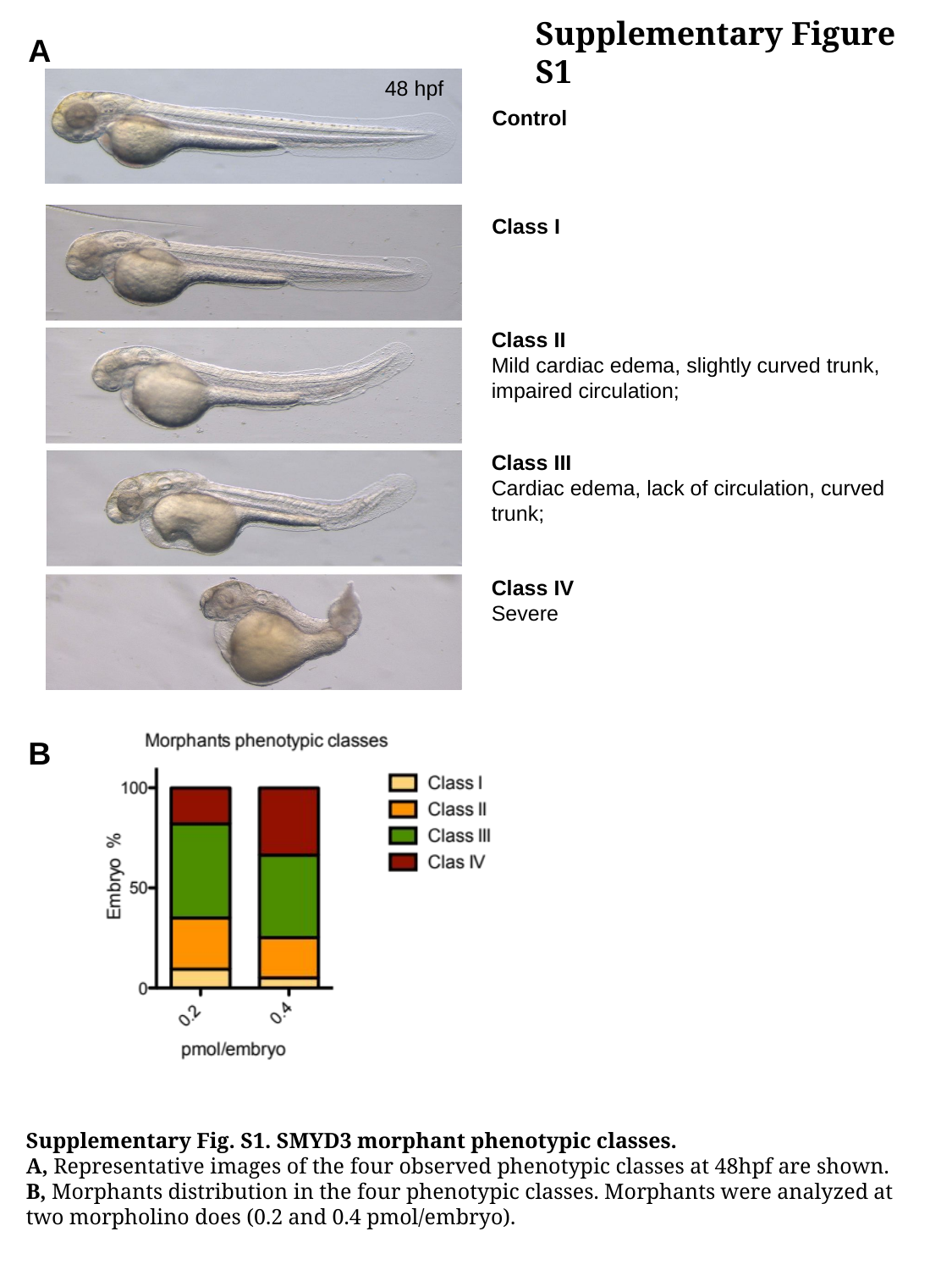

Supplementary Figure S1
A
48 hpf
Control
Class I
Class II
Mild cardiac edema, slightly curved trunk, impaired circulation;
Class III
Cardiac edema, lack of circulation, curved trunk;
Class IV
Severe
B
Supplementary Fig. S1. SMYD3 morphant phenotypic classes.
A, Representative images of the four observed phenotypic classes at 48hpf are shown.
B, Morphants distribution in the four phenotypic classes. Morphants were analyzed at two morpholino does (0.2 and 0.4 pmol/embryo).
